# Validation of the graded prognostic assessment for gastrointestinal cancers with brain metastases (GI-GPA)

**DOI:** 10.1186/s13014-020-1484-9

**Published:** 2020-02-13

**Authors:** Carsten Nieder, Mandy Hintz, Ilinca Popp, Angelika Bilger, Anca L. Grosu

**Affiliations:** 1 Department of Oncology and Palliative Medicine, Nordland Hospital, 8092 Bodø, Norway; 2 Department of Clinical Medicine, Faculty of Health Sciences, University of Tromsø, 9037 Tromsø, Norway; 3 Department of Radiation Oncology, University Hospital Freiburg, 79106 Freiburg, Germany; 4 German Cancer Consortium (DKTK), Partner Site Freiburg, Freiburg, Germany

**Keywords:** Brain metastases, Gastrointestinal cancer, Radiotherapy, Prognostic factors

## Abstract

**Purpose:**

The purpose of this study was to validate a new prognostic model (GI-GPA) originally derived from a multi-center database (USA, Canada, Japan).

**Patients and Methods:**

This retrospective study included 92 German and Norwegian patients treated with individualized approaches, always including brain radiotherapy. Information about age, extracranial spread, number of brain metastases, performance status and other variables was collected. The GI-GPA score was calculated as described by Sperduto et al.

**Results:**

Median survival was 4 months. The corresponding figures for the 4 different prognostic strata were 2.3, 4.4, 9.4 and 12.7 months, respectively (*p* = 0.0001). Patients whose management included surgical resection had longer median survival than those who were treated with other approaches (median 11.9 versus 3.0 months, *p* = 0.002). Comparable results were seen for additional systemic therapy (median 8.5 versus 3.5 months, *p* = 0.01).

**Conclusion:**

These results confirm the validity of the GI-GPA in an independent dataset from a different geographical region, despite the fact that overall survival was shorter in all prognostic strata, compared to Sperduto et al. Potential explanations include differences in molecular tumor characteristics and treatment selection, both brain metastases-directed and extracranially. Long-term survival beyond 5 years is possible in a small minority of patients.

## Introduction

According to recent data, survival of patients with brain metastases from gastrointestinal cancers has improved [1]. However, prognosis and eligibility for different treatment options varies with performance status, number of brain metastases and patterns of extracranial disease extent [2–7]. Given that brain metastases can occur early or late during the disease trajectory, management decisions are not always simple and straightforward [8, 9]. Despite progress, a median overall survival of 8 months [1] can still be considered disappointing.

Prognostic scores have long been used to support decision making and to stratify patients for research purposes [10, 11]. Models such as the graded prognostic assessment (GPA) [12, 13] have been validated in several studies and adopted by many clinicians. Recently, these tools have been updated to further refine their performance [14–16]. This is also true for the GPA for gastrointestinal primary tumors (GI-GPA) [1]. The latter 4-tiered score is based on Karnofsky performance status (KPS), age (cut-off 60 years), number of brain metastases and presence of extracranial metastases, while its predecessor solely reflected variations in KPS. The purpose of the present study was to validate the GI-GPA in an independent cohort of patients from Germany and Norway, hypothesizing that a validated score would gain wide acceptance.

## Material and methods

### Patients and treatment

A retrospective study based on chart review of 92 patients with irradiated brain metastases from GI cancers was performed. Patients managed with best supportive care rather than primary or post-operative radiotherapy were excluded. Treatment was individualized and consisted of focal therapies such as surgery, radiosurgery and stereotactic fractionated radiotherapy with or without whole-brain radiotherapy (WBRT), or upfront WBRT alone with total doses in the range of 20–40 Gy (5–20 fractions). According to the intention-to-treat principle, patients who failed to complete all fractions of radiotherapy were included in the study. Sequential salvage treatment of new or progressive intracranial lesions was individualized. All approaches mentioned above were considered at the time of relapse or progression. Systemic treatment before and after brain-directed measures was usually prescribed as judged appropriate by the patients’ medical oncologists. The patients (all-comers) were treated consecutively between 2005 and 2018 and identified from a previously described database [15–17], which includes data from the radiotherapy centers in Bodø and Freiburg. Prognosis was estimated on the basis of age, KPS, extracranial metastases and number of brain metastases as described in the recent GI-GPA publication [1] and shown in Table 1. Differences to the previous GPA score are also shown in the table.

**Table 1:** Baseline characteristics included in the GI-GPA (Sperduto et al. 2019 [1]): minimum point sum 0 (poor prognosis), maximum point sum 4 (good prognosis)

Parameter	GI-GPA	DS-GPA
Metastatic spread to extracranial sides	0	
Brain metastases only	0.5	
Age ≥60 years	0	
Age <60 years	0.5	
Karnofsky performance status ≤70	0	1 if 70
Karnofsky performance status 80	1	2
Karnofsky performance status 90-100	2	3 if 90, 4 if 100
Number of brain metastases >3	0	
Number of brain metastases 2-3	0.5	
Number of brain metastases 1	1	

*KPS* Karnofsky performance status

^a^includes patients with delayed (salvage) neurosurgery, radiosurgery, fractionated re-irradiation

### Statistical methods

Actuarial survival from the first day of radiotherapy or from surgery was calculated employing the Kaplan-Meier method, and different groups were compared using the log-rank test (SPSS 25, IBM Corp., Armonk, NY, USA). Date of death was known in all but 3 patients. The latter were included as censored observations after a median follow-up of 70 months. Uni- and multivariate Cox regression analysis was also performed (forward conditional method).

## Results

### Patient characteristics

The median age was 65 years (range 40–85). The median KPS was 70 (range 50–100). Most patients developed brain metastases late during the course of disease (median time interval after cancer diagnosis 26 months, range 0–143). The most common initial treatment approach was primary WBRT alone (53%), followed by surgery with or without post-operative radiotherapy (35%). The use of sequential systemic therapy was not well documented, except for 35 patients (19 received additional anti-cancer drugs while 16 did not). Further patient characteristics are shown in Table 2.

**Table 2:** Patient characteristics

Parameter	Number	Percent
Male gender	56	61
Female gender	36	39
Colon cancer	37	40
Rectal cancer	34	37
Esophageal cancer	14	15
Other GI cancer (gastric, pancreatic etc.)	7	8
Extracranial metastases	68	74
No extracranial metastases	24	26
Single brain metastasis	37	40
2–3 brain metastases	31	34
> 3 brain metastases	24	26
Age < 60 years	26	28
Age ≥ 60 years	66	72
KPS < 80	55	60
KPS 80	16	17
KPS 90–100	21	23
Upfront whole brain radiotherapy^a^	49	53
Upfront neurosurgery	32	35
Upfront radiosurgery	7	8
Upfront stereotactic fractionated radiotherapy	4	4

*KPS* Karnofsky performance status

^a^includes patients with delayed (salvage) neurosurgery, radiosurgery, fractionated re-irradiation

### GI-GPA

**Fig. 1:**
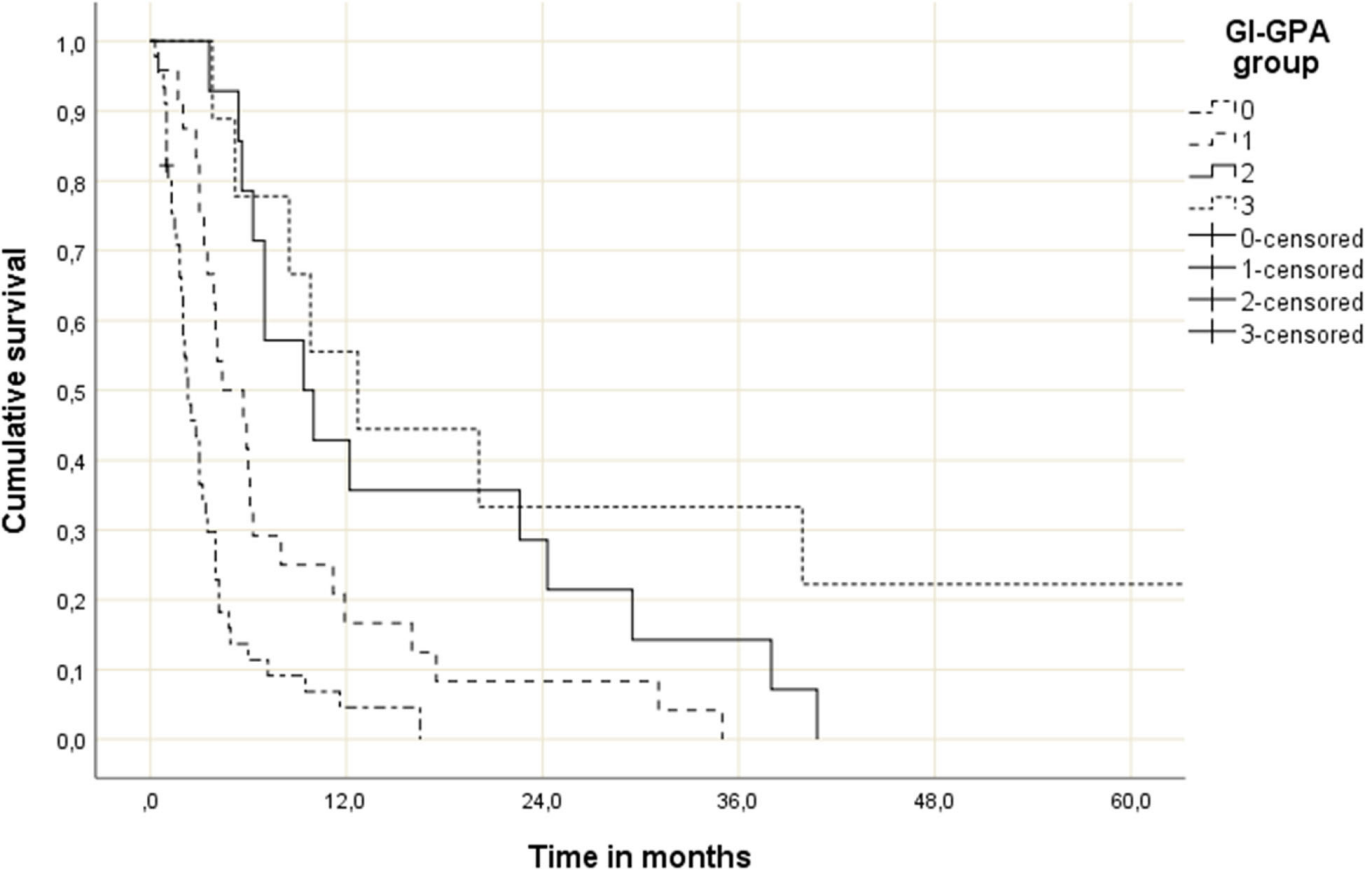
Actuarial survival of patients with GI cancer and brain metastases stratified by GI-GPA, *p* = 0.0001 (pooled over all strata)

Most patients had unfavorable prognostic features, i.e. 0–1 point in 45 patients (49%) and 1.5–2 points in 24 (26%). Fourteen patients (15%) had 2.5–3 points and the remaining 9 (10%) had 3.5–4 points. These four prognostic strata had significantly different median survival of 2.3, 4.4, 9.4 and 12.7 months (*p* < 0.0001, log-rank test pooled over all strata, Fig. 1). Overall median survival was 4.0 months. Table 3 shows the results of univariate prognostic factors for survival. In multivariate Cox regression analysis KPS (3 strata, *p* = 0.0001), number of brain metastases (3 strata, p = 0.0001) and extracranial metastases (2 strata, *p* = 0.04) were significant predictors of survival.

**Table 3:** Significant prognostic factors for overall survival (log-rank test or, for continuous variables, Cox regression)

Parameter	Median survival in months	*p*-value
Extracranial metastases	3.0	
No extracranial metastases	9.4	0.001
Single brain metastasis	8.5	
2–3 brain metastases	4.1	0.0001
More than 3 brain metastases	2.5	
Age < 60 years	4.9	
Age ≥ 60 years	4.0	0.6
Age as continuous variable		0.9
KPS as continuous variable		0.0001

*KPS* Karnofsky performance status

### Further survival results

Patients whose management included surgical resection had longer median survival than those who were treated with other approaches (median 11.9 versus 3.0 months, *p* = 0.002). Comparable results were seen for additional systemic therapy (median 8.5 versus 3.5 months, *p* = 0.01). Time interval to development of brain metastases was not prognostic. Regarding primary tumor site, the following median results were observed: esophageal cancer 5.4 months, rectal cancer 5.2 months, colon cancer 3.8 months, others 2.3 months (*p* = 0.18, log-rank test pooled over all strata). All patients who survived for 3 or more years had colorectal primary tumors. In the poor-prognosis group (0–1 points) median survival was 2.0 months after WBRT and 4.2 months after other approaches. The corresponding 1-year survival rate was 0 and 15%, respectively.

## Discussion

We report an independent validation study of the GI-GPA [1] in a European patient population, comparable to the previous validation of the Lung-molGPA [16] and the Melanoma-molGPA [15]. The study cohort consisted mainly of patients with poor or intermediate prognosis who were judged not to be appropriate candidates for aggressive local therapies, such as surgery or stereotactic radiotherapy. Nevertheless, all patients received active brain-metastases-directed therapy. Different treatment intensity and heterogeneity of gastrointestinal tumors (site, histology, molecular features) in part explains why the median survival in our study was 4 months, while the patients analyzed by Sperduto et al. [1] survived for a median of 8 months. Other treatments (chemotherapy, targeted drugs, salvage of brain metastases) might have differed too, however, they were not recorded in many of the patients. In the present study, additional systemic therapy was associated with significantly better survival (median 8.5 versus 3.5 months), consistent with a previous report [18]. Given that extracranial metastases were present in most patients (74%; Sperduto et al.: 79%), it is understandable that lack of extracranial disease control will negatively affect survival in this population. Although under continued investigation, systemic brain-directed therapy is uncommonly prescribed [19].

Median time interval from initial cancer diagnosis to brain metastases was 26 months (Sperduto et al.: 23 months), which represents a longer interval than in other primary tumors, e.g. lung cancer [8, 12]. Our study population was heavily weighted towards colorectal primary tumors (77%; Sperduto et al.: 54%). Sperduto et al. reported that primary tumor site significantly influenced survival, although this variable was not included in the GI-GPA score. The same was true for serum hemoglobin. Other reports have also suggested that patients with gastric or pancreatic cancer constitute a minority of GI cancer patients who develop brain metastases [20, 21]. Thirty-one percent of the patients analyzed by Sperduto et al. [1] had KPS 90–100, compared to only 23% of our patients. Interestingly, survival was shorter in our study even for each different GI-GPA group (Table 4). Besides explanations discussed earlier, this could also result from differences in the diagnostic setting (imaging in asymptomatic patients vs. clinical deficits), causing a potential lead time bias if the patients in the Sperduto et al. cohort were treated earlier. In principle, the difference in patient numbers (92 compared to 792) may have contributed to different results, too. It would be interesting to see additional studies in patients managed with different approaches in different regions of the world.

**Table 4:** Survival outcomes stratified by study

Group	Median survival in months	6-month probability^a^	12-month probability^a^
0–1 p.	2.3	11%	5%
Sperduto et al. 0–1 p.	3	30%	14%
1.5–2 p.	4.4	38%	13%
Sperduto et al. 1.5–2 p.	7	53%	37%
2.5–3 p.	9.4	71%	36%
Sperduto et al. 2.5–3 p.	11	67%	47%
3.5–4 p.	12.7	78%	56%
Sperduto et al. 3.5–4 p.	17	87%	68%

^a^estimated from [1] Fig. 1

The main result of our study was that the GI-GPA accurately reflects the prognostic impact of different baseline characteristics, although we did not see a significant impact of age (possibly due to the difference in statistical power). In the study reported by Sperduto et al. discrimination between the two favorable groups was better than in ours, either, and most likely, due to the larger numbers of patients or the variable impact of age in the two studies. When looking at both studies together, the GI-GPA score seems to represent a useful improvement of its ancestors such as DS-GPA [12, 22]. Age, KPS and number of brain metastases were also part of a previously published nomogram (227 patients from Italy, all with colorectal cancer) [11].

Limitations of this study, which followed the methods used in previous validation studies [15, 16], include the small number of patients, which were recruited over a long period of more than 10 years, statistical power of subgroup analyses, and retrospective design. Given that patients managed with best supportive care were excluded, worse survival outcomes could be expected if one would analyze all patients with a brain metastasis diagnosis. In selected patients survival beyond 5 year was observed, in line with Sperduto et al. [1] and other authors [23–26]. These results lend support to the current clinical practice of surgical resection and/or ablative radiotherapy for oligometastatic lesions. The results also confirm the limited median survival after primary WBRT reported previously [27]. Sperduto et al. [1] found median survival of 3 months, which is identical to the present analysis. They suggested that best supportive care may be considered if the GI-GPA indicates a poor prognosis (0–1 points). In our poor-prognosis group median survival was 2.0 months after WBRT and 4.2 months after other approaches. The corresponding 1-year survival rate was 0 and 15%, respectively. Thus, individual assessment and multi-disciplinary decision making is recommended to identify those patients who might benefit from active therapy.

## Conclusions

The data presented in this study confirm the validity of the GI-GPA in patients from a different geographical region. However, median survival was shorter in all prognostic strata. Potential explanations include differences in treatment selection, both brain metastases-directed and with systemic agents. Long-term survival beyond 5 years is possible in a small minority of patients.

## Data Availability

The dataset supporting the conclusions of this article is available at request from the corresponding author, if intended to be used for meta-analyses.

## Abbreviations

DS-GPA: Disease-specific graded prognostic assessment
GI: Gastrointestinal
GI-GPA: Graded prognostic assessment for gastrointestinal primary tumors
GPA: Graded prognostic assessment
KPS: Karnofsky performance status
molGPA: Molecular markers in graded prognostic assessment
WBRT: whole-brain radiotherapy
